# Association between age at menarche and risk of myopia in the United States: NHANES 1999–2008

**DOI:** 10.1371/journal.pone.0285359

**Published:** 2023-05-05

**Authors:** In Jeong Lyu, Sei Yeul Oh

**Affiliations:** 1 Department of Ophthalmology, Korea Cancer Center Hospital, Korea Institute of Radiological and Medical Sciences, Seoul, Republic of Korea; 2 Department of Ophthalmology, Samsung Medical Center, Sungkyunkwan University School of Medicine, Seoul, Korea; National Yang-Ming University Hospital, TAIWAN

## Abstract

We evaluate the effect of menarche on myopia in women in the United States (US). A cross-sectional survey and examination were conducted using data from the 1999–2008 US National Health and Nutrition Examination Survey (NHANES), and 8,706 women aged ≥20 years (95% confidence interval [CI], 44.23 to 45.37) were enrolled. Characteristics were compared between nonmyopic and myopic participants. Univariable and multivariable logistic regression analysis was performed to evaluate the risk factors for myopia. A minimum p-value approach was used to estimate the cut-off point for age at menarche. The prevalence of myopia was 32.96%. Mean spherical equivalent (SE) were -0.81 diopters (95% CI, -0.89 to -0.73) and the mean age of menarche was 12.67 years (95% CI, 12.62 to 12.72). In the crude logistic regression model, age (odd ratio [OR] 0.98), height (OR, 1.02), astigmatism (OR, 1.57) (all p < 0.0001), age at menarche (OR, 0.95; p = 0.0005), white ethnicity, being born in the US, higher level of education, and higher annual household income (all p trend <0.0001) were significantly associated with myopia. 1-year increments in age at menarche was associated with a 4% decrease in the risk of myopia after adjusting for age, height, body mass index (BMI), ethnicity, and astigmatism (OR, 0.96; 95% CI, 0.93 to 0.99, p = 0.0288). The cut-off value for age at menarche was 15 years by the maximum chi-square test (p < 0.0001). Age at menarche may attribute to myopia progression, along with other environmental and individual risk factors.

## Introduction

Myopia is the most common ophthalmologic problem that starts in childhood [1–3]. Many factors are involved in its progression. Well-known risk factors for myopia are ethnicity [4], higher level of education [5–7], minimal outdoor activity [6, 8], excessive close work [6, 9], and parental myopia [6, 9, 10]. However, several reports suggest that the timing of puberty is associated with myopia [11–13]. We previously found that a later age at menarche is associated with a decreased risk of myopia in Korean adults, based on data obtained from the Korean National Health and Nutrition Examination Survey (KNHANES) [11]. In addition, a nationwide cross-sectional study in China evaluated the association between menarche status and myopia [12]. The study included girls aged 10–15 years and demonstrated that menarche onset was associated with risk of myopia [12]. Another 2-year observational study showed that changes in estradiol levels were associated with refractive and axial length changes in Chinese children [13].

Based on this perspective, evaluating the influence of age at menarche on myopia in other countries with different ethnicities and prevalence of myopia is meaningful. Hence, we evaluated the effect of age at menarche on myopia by using the data of the National Health and Nutrition Examination Survey (NHANES) in the United States (US).

## Methods

### Study participants

**Fig 1:**
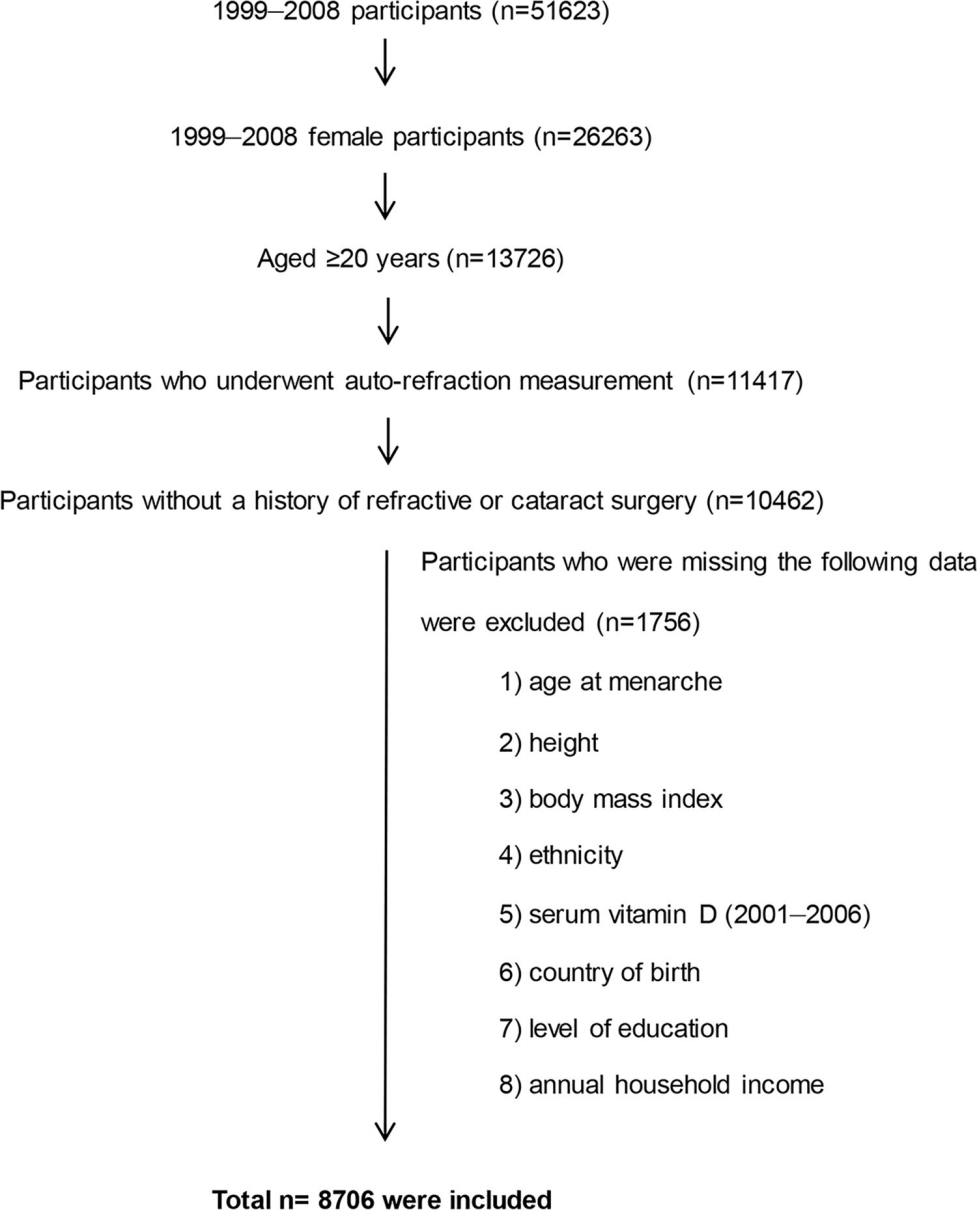
Flow chart of paticipant inclusion and exclusion from the National Health and Nutrients Examination Survey in this study.

The NHANES (www.cdc.gov/nchs/nhanes.htm) is a national representative survey conducted by the National Center for Health Statistics, Centers for Disease Control and Prevention (CDC). We used data from the 1999–2008 US NHANES, which included ophthalmic evaluation. From 1999 to 2008, 51,623 subjects participated in the health interview and examination survey, including 13,726 women aged 20 years and above. Among them, 11,417 participants had undergone non-cycloplegic autorefraction of the right eye. Then, we excluded 955 subjects who had a previous history of refractive or cataract surgery, which can considerably influence the refractive error. Participants with missing data on age at menarche, height, body mass index (BMI), ethnicity, serum vitamin D level (from 2001 to 2006), country of birth, level of education, and annual household income were excluded from this study (n = 1756). Finally, data of 8,706 female participants, representative of 76,914,516 US women, were analyzed. Flowchart of participant selection is shown in Fig 1.

This study adhered to the tenets of the Declaration of Helsinki. It was approved by the Institutional Review Board (IRB) of Samsung Medical Center (IRB no. SMC 2016-01-005). The requirement for informed consent from individual patients was waived because data used were public and anonymized under confidentiality guidelines.

### Examination and variable definitions

The Nidek Auto Refractor Model ARK-760 (Nidek Co Ltd, Gamagori, Japan) was employed to measure the refractive error and the median value of three objective measurements—sphere, cylinder, and axis for both eyes, after removing the usual corrective lenses. The spherical equivalent (SE) refractive error was calculated as the sphere + 1/2 cylinder. Myopia was defined as a SE less than or equal to -1.0 diopters (D) according to the previous studies of refractive errors using NHANES [4, 14].

The NHANES are prospectively collected household questionnaire-based studies. Households were selected using a stratified, randomized, multistage, probability-cluster design. Health interviews were conducted in the participant’s home in either English or Spanish. Demographics and socioeconomic factors included age, height, BMI, ethnicity, country of birth, level of education, and annual household income. BMI was calculated as body weight (kg)/height (m)^2^. Ethnicity was categorized as white, Hispanic, black, and others. Participants responded to an open-ended question about the age at menarche: “How old were you when you had the first menstrual period?” Country of birth was categorized into three categories as born in the US, Mexico, or elsewhere. Participants also responded to a question about the level of education “What is the highest grade or level of school that you have completed or the highest grade you have received?” Educational level was divided into three groups: less than high school graduate, high school graduate or equivalent, and more than high school graduate. Annual household family income was divided into quartiles by each cycle of data. Serum vitamin D level was obtained by measuring the serum concentration of 25(OH)D, performed in NHANES 2001–2006. Therefore, serum vitamin D level was analyzed separately.

### Statistical analyses

The SE values in the right and left eyes were highly correlated in Pearson’s correlation analysis (r = 0.939, p < 0.0001). Therefore, we used the right eye for the analysis in this study. Survey weights were calculated to account for the survey year, stage of selection, and non-response.

To evaluate the risk factors for myopia, we conducted logistic regression analyses with “nonmyopia” as the reference level. Age, height, BMI, ethnicity, and astigmatism, which can be confounders between age at menarche and myopia, were adjusted in multivariable model. P values and 95% CI (confidence interval) for OR (odds ratio) were calculated, and p values were adjusted using Bonferroni’s method for multiple comparisons was applied to the p-values. We used the maximum chi-square test to choose the optimal cut-off value.

All p values were two-sided, and p values less than 0.05 were considered statistically significant. Statistical analyses were performed using SAS version 9.3 (SAS Institute, Cary, NC, USA).

### Results

Among the 8,706 female participants, representative of 76,914,516 US women, the weighted prevalence of myopia was 32.96%. The mean SE and astigmatism were -0.81 D (95% CI, -0.89 to -0.73) and 0.8 DC (cylinder diopters) (95% CI, 0.75 to 0.83), respectively. The characteristics of the 8,706 participants are summarized in Table 1. The most common ethnicity was white (71.98%), followed by Hispanic (11.77%), Black (11.24%), and other (5.01%). The median age of the study participants was 44.80 years (95% CI, 44.23 to 45.37). The mean height was 162.67 cm (95% CI, 162.47 to 162.87) and BMI was 28.48 kg/m^2^ (95% CI, 28.23 to 28.74). The mean age of menarche was 12.67 years (95% CI, 12.62 to 12.72). The mean vitamin D level was 62.39 nmol/L (95% CI, 60.69 to 64.09). Among the participants, 58.18% had an more than high school graduate. About 87% participants were born in the US.

**Table 1:** Characteristics of the study population according to the spherical equivalent (n = 8706).

Variables	Nonmyopia (n = 6143)	Myopia (n = 2563)	Total (n = 8706)
Spherical equivalent, diopters	+0.51 (0.47, 0.55)	-3.50 (-3.62, -3.37)	-0.81 (-0.89, -0.73)
Astigmatism, cylinder diopters	0.72 (0.70, 0.74)	0.98 (0.93, 1.02)	0.80 (0.78, 0.83)
Age, years	46.43 (45.79, 47.08)	41.47 (40.74, 42.19)	44.8 (44.23, 45.37)
Height, cm	162.36 (162.12, 162.6)	163.3 (163.03, 163.57)	162.67 (162.47, 162.87)
BMI, kg/m^2^	28.58 (28.31, 28.85)	28.28 (27.88, 28.68)	28.48 (28.23, 28.74)
Ethnicity/Race			
Hispanic	11.82 (9.73, 13.91)	10.07 (8.27, 11.86)	11.24 (9.37, 13.11)
White	70.16 (66.85, 73.48)	75.67 (72.81, 78.53)	71.98 (69.09, 74.87)
Black	13.25 (10.82, 15.69)	8.76 (7.10, 10.41)	11.77 (9.74, 13.81)
Others	4.76 (3.82, 5.70)	5.50 (4.21, 6.80)	5.01 (4.19, 5.82)
Age at menarche, years	12.72 (12.66, 12.78)	12.58 (12.51, 12.65)	12.67 (12.62, 12.72)
Vitamin D level, nmol/L^a^	61.96 (60.21, 63.71)	63.23 (61.06, 65.39)	62.39 (60.69, 64.09)
Country of birth			
US	84.97 (82.71, 87.24)	91.03 (89.43, 92.63)	86.97 (85.15, 88.79)
Mexico	4.39 (3.60, 5.18)	2.00 (1.62, 2.38)	3.60 (3.03, 4.18)
Elsewhere	10.64 (8.64, 12.63)	6.97 (5.44, 8.50)	9.43 (7.78, 11.08)
Level of education			
<High school graduate	19.57 (18.01, 21.13)	10.82 (9.39, 12.25)	16.69 (15.39, 17.99)
High school graduate	26.91 (25.42, 28.39)	21.51 (19.32, 23.69)	25.13 (23.84, 26.41)
>High school graduate	53.52 (51.34, 55.70)	67.67 (64.98, 70.37)	58.18 (56.26, 60.11)
Household income			
Low (1^st^)	25.89 (24.16, 27.62)	18.70 (16.51, 20.89)	23.52 (21.96, 25.07)
Mid-low (2^nd^)	27.18 (25.51, 28.85)	22.41 (19.85, 24.96)	25.61 (23.93, 27.28)
Mid-high (3^rd^)	24.90 (23.46, 26.34)	27.52 (25.00, 30.05)	25.76 (24.42, 27.10)
High (4^th^)	22.03 (20.02, 24.05)	31.37 (28.26, 34.49)	25.11 (23.04, 27.18)

The data are presented as the mean (95% confidence interval).

^a^The serum vitamin D level was measured only in NHANES 2001–2006.

BMI, body mass index; US, United States.

### Risk factors associated with myopia

In the crude logistic regression model, age (OR, 0.98; 95% CI, 0.98 to 0.98; p < 0.0001), height (OR, 1.02; 95% CI, 1.01 to 1.03; p < 0.0001), astigmatism (OR, 1.57; 95% CI, 1.44 to 1.71; p < 0.0001), age at menarche (OR, 0.95; 95% CI, 0.92 to 0.98; p = 0.0005), white ethnicity (p trend < 0.0001), being born in the US (p trend < 0.0001), higher level of education (p trend < 0.0001), and higher annual household income (p trend < 0.0001) were significantly associated with myopia. In a model adjusted for age, height, BMI, ethnicity, and astigmatism, older age at menarche (OR, 0.96; 95% CI, 0.93 to 0.99; p = 0.0288), being born in the US (p trend <0.0001), higher level of education (p trend <0.0001), and higher annual household income (p trend <0.0001) also remained significant (Table 2). However, when the country of birth, level of education, and household income were additionally adjusted in multivariable model, the statistical significance of older age at menarche decreased (OR, 0.97; 95% CI, 0.97 to 1.00; p = 0.086).

**Table 2:** Risk factors affecting myopia.

	Univariable model		Multivariable model^a^	
	OR (95% CI)	p-value	OR (95% CI)	p-value
Astigmatism, cylinder diopters	1.57 (1.44, 1.71)	**<0.0001**		
Age, years	0.98 (0.98, 0.98)	**<0.0001**		
Height, cm	1.02 (1.01, 1.03)	**<0.0001**		
BMI, kg/m^2^	0.99 (0.99, 1.00)	0.1596		
Ethnicity/Race^c^		**<0.0001**		
Hispanic	1.00 (reference)			
White	1.63 (1.37, 1.94)			
Black	1.29 (1.07, 1.55)			
Others	1.75 (1.26, 2.43)			
Age at menarche, years	0.95 (0.92, 0.98)	**0.0005**	0.96 (0.93, 0.99)	**0.0288**
Vitamin D level, nmol/L	1.00 (1.00, 1.01)	0.1691		
Country of birth^b^		**<0.0001**		**<0.0001**
US	1.00 (reference)		1.00 (reference)	
Mexico	0.42 (0.34, 0.53)		0.48 (0.34, 0.67)	
Elsewhere	0.61 (0.49, 0.77)		0.64 (0.49, 0.84)	
Level of education^b^		**<0.0001**		**<0.0001 **
<High school graduate	1.00 (reference)		1.00 (reference)	
High school graduate	1.45 (1.22, 1.72)		1.33 (1.08, 1.64)	
>High school graduate	2.29 (1.95, 2.68)		2.00 (1.63, 2.46)	
Household income^c^		**<0.0001**		**<0.0001**
Low (1^st^)	1.00 (reference)		1.00 (reference)	
Mid-low (2^nd^)	1.14 (0.96, 1.36)		1.20 (0.97, 1.48)	
Mid-high (3^rd^)	1.53 (1.28, 1.83)		1.60 (1.28, 2.01)	
High (4^th^)	1.97 (1.65, 2.35)		2.12 (1.69, 2.65)	

^a^Age, height, BMI, ethnicity, and astigmatism were adjusted.

OR, odds ratio; CI, confidence interval.

A value of p < 0.05 is statistically significant and is indicated in bold font.

^b^The p value was corrected by using Bonferroni correction for multiple testing (multiplied by 2).

^c^The p value was corrected by using Bonferroni correction for multiple testing (multiplied by 3).

### Age at menarche and myopia

**Fig 2:**
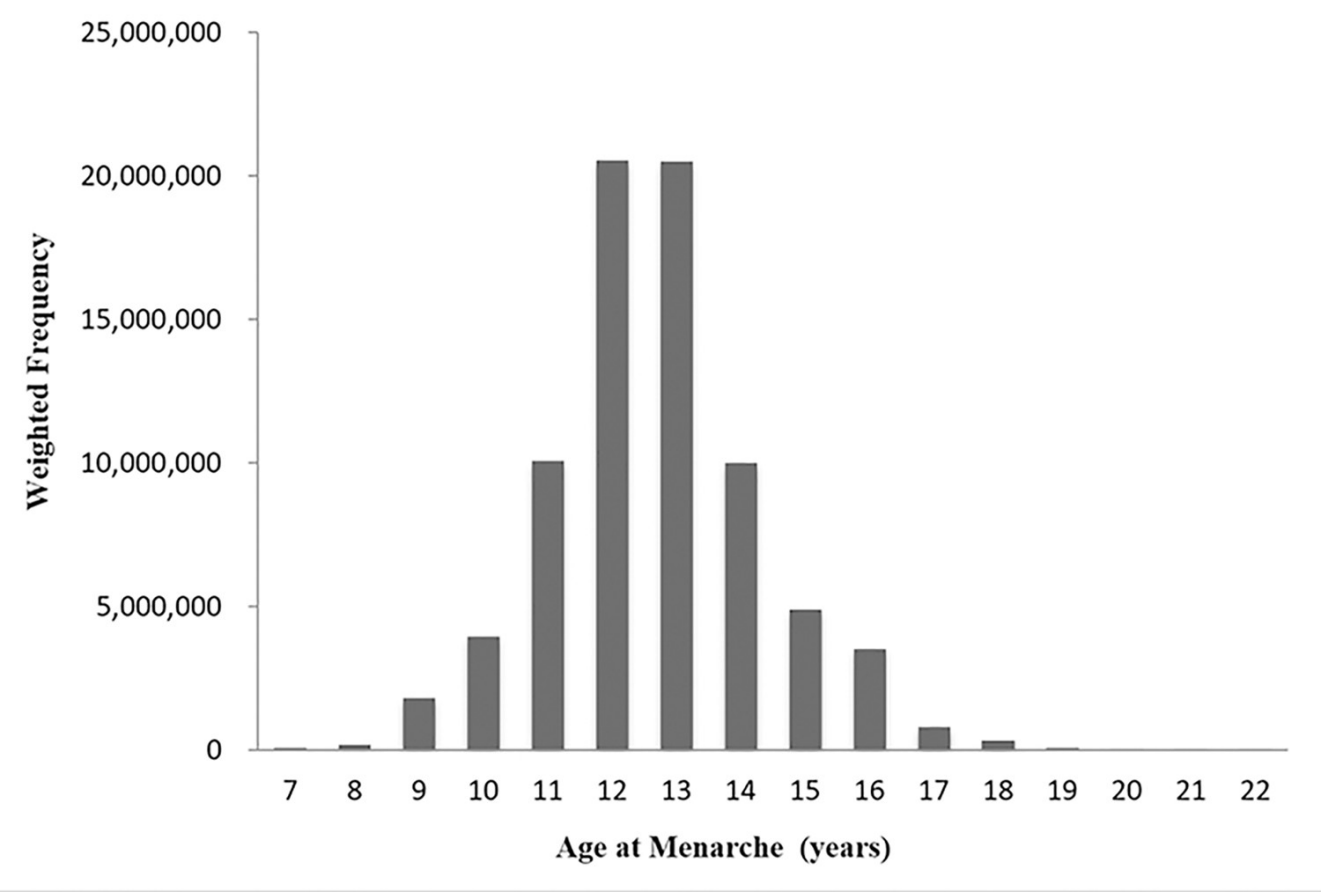
Distribution of age at menarche among the participants of the National Health and Nutrients Examination Survey.

The distribution of the age at menarche is presented in Fig 2. The mean age at menarche was 12.72 years (95% CI, 12.66 to 12.78) in the nonmyopia group and 12.58 years (95% CI, 12.51 to 12.65) in the myopia group (p = 0.0005).

**Fig 3:**
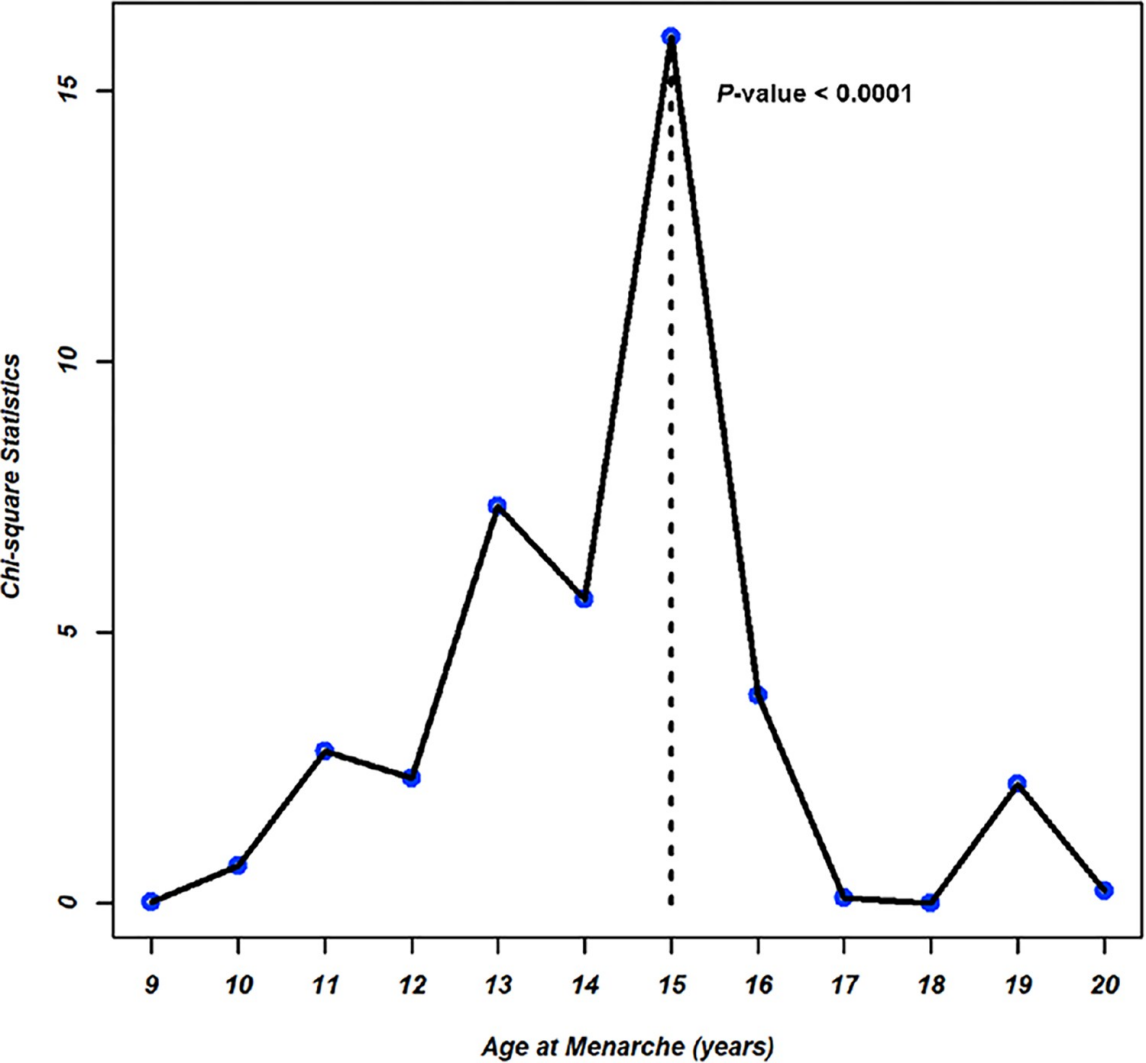
Optimal cut-off point displayed along with the different cut-off points with the corresponding p values plotted on a logarithmic scale. The optimal cut-off point was 15 years in National Health and Nutrients Examination Survey (p < 0.0001).

In the univariable logistic regression model, each year increase in age at menarche was associated with a 5% decrease in the risk of myopia (OR, 0.95; 95% CI, 0.92 to 0.98, p < 0.0005). After adjustment for age, height, BMI, ethnicity, and astigmatism, 1-year increments in age at menarche was associated with a 4% decrease in the risk of myopia (OR, 0.96; 95% CI, 0.93 to 0.99, p = 0.0288). The optimal cut-off value of age at menarche as a risk factor for myopia was established as 15 years (p < 0.0001) (Fig 3).

## Discussion

The NHANES was designed to assess the health and nutritional status of adults and children. Ocular examinations were conducted between 1999 and 2008. The aim of the present study was to evaluate the risk factors for myopia and to clarify the relationship between age at menarche and myopia in the US. Age at menarche was revealed as a potential risk factor for myopia, along with other risk factors such as age, height, ethnicity, country of birth, level of education, and annual household income. After adjusting for height, weight, and ethnicity, each 1-year increase in age at menarche was associated with a 4% decrease in the risk of myopia.

Menarche is one of the most important indicators of puberty in girls [15]. Therefore, hormonal changes during puberty may explain the association between age at menarche and myopia. Estrogen is involved in ocular growth by regulating the activity of the matrix metalloproteinases (MMPs) and the nitric oxide signal cascade in the retinal pigment epithelium and the scleral extracellular matrix [16–18]. Estrogen also modulates the biochemical properties and curvature of the cornea [19]. Wang et al. [13] demonstrated that the change in estradiol level is significantly associated with axial length elongation and decreased SE, along with parental myopia and short outdoor time in Chinese children. Their report also demonstrated that the protective effect of outdoor time on myopia was stronger in girls before menarche. Wang et al. [13] propose that puberty may have a regulatory role on outdoor time on myopia. Otherwise, the increased level of insulin-like growth factor-1 (IGF-1) during puberty may have other key roles on refractive changes [20]. The surge in growth hormone secretion induces increased IGF-1 levels [21, 22]. IGF-1 in scleral fibroblasts contributes to axial elongation [20, 23]. However, the exact mechanism of how age at menarche impacts myopia remains unknown. In this study, in the multivariable adjusted model with age, height, BMI, and astigmatism, the age at menarche significantly affected myopia. Nevertheless, the significance of age at menarche deceased when the country of birth, level of education, and household income were additionally adjusted. Menarche, which represents the onset of puberty, could have a complex involvement in the development of myopia via interacting with other demographic and environmental risk factors. Therefore, further studies are required to determine the actual effect of menarche on development of myopia.

This study has several limitations. First, this study had a cross-sectional design, which limits assigning causality. Recall bias may also exist because of the self-reporting of menarche. Previously validity of age at menarche recalled was evaluated in several studies and presented correlation coefficients of 0.67 [24] and 0.79 [25]. However, in 2007, Cooper et al. [26] reported the agreement of age at menarche between middle aged compared with recorded in adolescence was moderate (**κ** = 0.30) and coefficients was 0.59. They suggest that age at menarche self-reported in middle age is influenced by educational level and having experienced a miscarriage or stillbirth, and not very accurate. Therefore, it should be aware that recall bias may affect the interpretation of the results analyzed using menarche age, especially in studies including middle and old aged participants. Further well-designed prospective study will be needed to prove the exact impact of onset of age at menarche on myopia.

Second, several potential risk factors were not included in this study because these data were lacking in the NHANES. These factors were parental history of myopia, age of onset of myopia, history of retinopathy of prematurity, history of near work, and time spent outdoors in childhood. These factors could be potential confounders or risk factors for myopia. We attempted to correct for time spent outdoors by analyzing vitamin D levels. However, vitamin D levels can vary seasonally and are only an indicator of recent time spent outdoors; thus, vitamin D levels are unsuitable for use as a marker of time spent outdoors in older adults participating in myopia studies [27]. Third, data on axial length could not be analyzed together with refractive error because examination of the axial length was not included in the NHANES. Nevertheless, to the best of our knowledge, this study appears to be the first to analyze the relationship between age at menarche and myopia by using nationally representative US data.

In conclusion, older age at menarche significantly decreased the risk of myopia according to an age, height, BMI, ethnicity, and astigmatism adjusted model. Menarche earlier than 15 years of age was a risk factor for myopia. These results support the findings of a previous study regarding the association between age at menarche and myopia.
